# Canine Rabies in India

**Published:** 1896-11

**Authors:** 


CANINE RABIES IN INDIA. Mr. J. C. Vaughan, of West Bengal, contributes a paper on this subject to the *Indian Medical Gazette* for August, in which he gives a detailed account of his experience with rabies in his own dogs, the main points of which are as follows: In every case, he says, the first thing which aroused his suspicions was an excess of freshness, a greater show than usual of affection for their master, anp a somewhat increased display of vigor and vitality in all they did, with a slight nervous excitability; but there was no irritability or snapping at other dogs. There was, perhaps, a tendency to bark too much for no ostensible reason. The preliminary catarrh, of which the veterinary surgeon Mr. Martin speaks, did not occur in these dogs. The suspicioussy mptoms never proved to be a false alarm in the author's experience. The excitability showed a steady tendency to increase, and within twenty-four hours the dog's eyes became dull and there was a half-drugged, far-away expression in the face and eyes which was strangely out of keeping with the still restless and excitable manner. At this stage the food was eaten with increased greed and haste; there was still the curious, isolated, purposeless bark, which was distinctly changed from the sound of the full bark. This voice-condition was the first symptom of the weakening of the muscular innervation of the pharyngo-laryngeal region, which, says the author, is so distinctive of hydrophobia. At this time also a frequent movement of the tongue was noticed.

Gradually the excited manner quieted down, although occasionally the old excitement broke out and the eyes flashed with a dull light. In the next period of the affection there were tremors of the limbs, subsultus tendinum of all the legs, and a quivering in the lower jaw; there was a troubled sleep, from which the dogs awoke more restless than ever. They were now quite mad, says Mr. Vaughan. The excitability, which had returned, the dull look in the eyes, the dilated pupils, the staring coat, and the slight stagger of the hindquarters which had set in, made up a picture which it was impossible to mistake. The dogs refused solid food, the temperature rose, the urine was high-colored, and the feces were almost black. Though they were still docile, continues Mr. Vaughan, and glad to receive their masters or visitors with expression of affection, it was at this time that they became uncertain.

In this condition, he says, their jaws may snap together in the excited affection they show, and they bite without meaning to. From this time they seem gradually to lose self-control and become more and more dangerous. Even at their very worst, the author states that his dogs never altogether lost their self-control, and that he was able to go up to them, carefully gloved, and pat them, and, although they snapped their jaws at him, it was unintentional, as their manner showed. The paralysis of the hindquarters and of the pharyngo-larnygeal region increases at this stage, and the period of excitement passes more or less quickly into one of deepening stupor, from which there are often convulsive awakenings; the intervals between these grow longer, and finally death ensues.

The author states that he watched these cases very carefully to the end, and it is very clear, he thinks, that the disease described is the form known as furious rabies. Dumb rabies begins very much in the same way; the restless sleep seems to be the turning-point, and the dog wakes up paralyzed, with dumb rabies or the furious form. A very dangerous period in the disease, Mr. Vaughan thinks, is the preliminary stage, just before the expression changes, for the nature of the case is then so hard to recognize.

A very important point is the question of the immediate treatment of bites from rabid dogs or doubtful ones. The author has always practised washing the wound freely at once and then burning it, not with solid silver nitrate, as is the usual way, but with strong fuming nitric acid or with strong hydrochloric acid.

Even in the case of bites from rabid dogs this plan, he states, has effectually prevented hydrophobia. This treatment was carried out in the case of a relative of the author, who was bitten by a rabid dog twenty-four years ago, with a successful result, for the man is still living. On one occasion several dogs were bitten by a rabid dog; this treatment was at once instituted and none of the dogs died of hydrophobia. Mr. Vaughan thinks that the same trust cannot be placed in nitrate of silver, for the following reason: When it is a question of a deep bite from the long canine fang of a dog, the tooth, which is a blunt instrument, has been driven by main force through the skin into the tissues, and, when so driven in, was coated with the poison-containing saliva, which was forced into the intercellular spaces of the tissue penetrated by the tooth, and although some of it lies in the wound-cavity or lines the edges of the wound-cavity inside, some or most of it has been jammed into the intercellular spaces around the cavity and lies deeper in the tissues. The wound-cavity in the meantime is filled with it and its sides moistened with serum-containing albumin. The nitrate of silver stick now penetrates into the wound-cavity, reaches the saliva and serum lining its sides, kills the lining-cells, and coagulates all the albumin within reach. The albumin-film thus formed makes, no doubt, says the author, a film which only protects the deeper-lying saliva and its poison; and in a deep, penetrating bite, forcing in a stick of nitrate of silver is very much like repeating the bite, and serves to drive the deeper-lying saliva only deeper still into the tissues and so to place it further outside, hence better protected by the albumin-coagulum film formed in the wound by the nitrate of silver treatment. If no albumin was coagulated in the wound, the caustic would or might reach the furthest-off and most deeply lying saliva. But as nitrate of silver forms an albumin coagulum, why not use, asks the author, an equally strong caustic which forms no coagulum? Hence the use of the fuming acid, which dissolves all albumin it reaches and penetrates at once, without the use of force, into all the surrounding tissue-spaces. The same holds good for all kinds of bites, and the penetrating acid kills all it reaches. One or two drops suffice for each bite, the slough soon separates, and the clean wound then left heals readily.

Mr. Vaughan states that he has treated a great many bites in this way, and when his own dogs went mad he invariably found that they had been bitten while he was away from home.

For the preceding reasons he says that he has always strongly objected to cauterizing any bites with nitrate of silver, and has invariably advocated the use of strong acid. But whatever the immediate treatment is, it must be prompt.

